# Ammonium chloride catalyzed synthesis of novel Schiff bases from spiro[indoline-3,4′-pyran]-3′-carbonitriles and evaluation of their antimicrobial and anti-breast cancer activities

**DOI:** 10.1186/s40064-016-2458-0

**Published:** 2016-06-24

**Authors:** Hossa F. Al-Shareef, Heba A. Elhady, Amany H. Aboellil, Essam M. Hussein

**Affiliations:** Department of Chemistry, Faculty of Applied Sciences, Umm Al-Qura University, Makkah, Saudi Arabia; Department of Chemistry, Faculty of Science (Girl’s), Al-Azhar University, Cairo, Egypt; Department of Botany and Microbiology, Faculty of Science, Cairo University, Giza, Egypt; Department of Chemistry, Faculty of Science, Assiut University, Assiut, 71516 Egypt

**Keywords:** Ammonium chloride, Schiff bases, Spiro[indoline-3,4′-pyran]-3′-carbonitriles, Antimicrobial, Anti-breast cancer

## Abstract

**Background:**

Indolinone and spiro-indoline derivatives have been employed in the preparation of different important therapeutic compounds required for treatment of anticonvulsants, antibacterial, Antitubercular, and anticancer activities. Schiff bases have been found to possess various pharmacological activities such as antitubercular, plant growth inhibiting, insecticsidal, central nerve system depressant, antibacterial, anticancer, anti-inflammatory, and antimicrobial. Mannich bases have a variety of biological activities such as antibacterial and antifungal activities.

**Results:**

In this study, a green, rapid and efficient protocol for the synthesis of a new series of Schiff bases from spiro[indoline-3,4′-pyran]-3′-carbonitrile derivatives using ammonium chloride as a very inexpensive and readily available reagent. The prepared compounds were assessed in vitro for their antimicrobial activity. Also, the cytotoxic activity of the prepared compounds was assessed in vitro against human cells line MCF7 breast cancer.

**Conclusion:**

Good activity was distinguished for Schiff bases from spiro[indoline-3,4′-pyran]-3′-carbonitriles, with some members recorded higher antimicrobial and anti-breast cancer activities.

**Graphical abstract:**
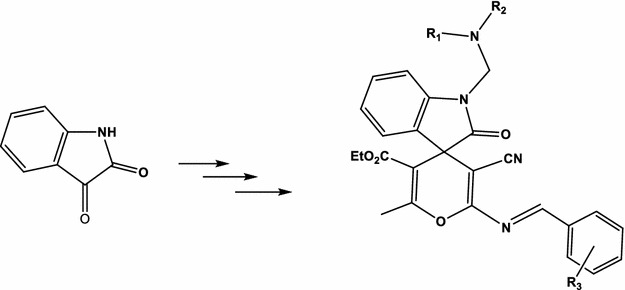
Novel Schiff bases from spiro[indoline-3,4′-pyran]-3′-carbonitriles

## Background

The development of eco-friendly and environmentally benign catalytic systems is one of the main themes of modern organic synthesis. Ammonium chloride (NH_4_Cl) is a very inexpensive and readily available catalyst; it has been reported as a catalyst for the synthesis of various heterocyclic compounds (Shaabani et al. 2003; Dabiri et al. 2009; Fortenberrya et al. 2013; Foroughifarab et al. 2011; Maleki and Salehabadi 2010; Shaabani et al. 2008; Hussein 2015). There are many bioactive molecules which possess various heteroatoms such as nitrogen, sulfur and oxygen, always taken the attention of chemists over the years mainly because of their biological significance. Pyrano derivatives have well-known biological effects, such as analgesic and anti-inflammatory activities (El-Zohry et al. 2008a). Indolinone and spiro-indoline derivatives have possessed broad-spectrum therapeutic activities such as anticonvulsants (Ragavendran et al. 2007; Azam et al. 2009; Sridhar et al. 2002), antibacterial (Rahman et al. 2004; Singh and Luntha 2009; Olomola and Bada 2009), Antitubercular (Sriram et al. 2005), and anticancer activities (Vine et al. 2007; Solomon et al. 2009; Wee et al. 2009). Schiff bases are important compounds owing to their wide range of biological activities and industrial applications. They have been found to possess various pharmacological activities such as antitubercular (Kascheres 2003), plant growth inhibiting (Simunek and Machácek 2010), insecticsidal (Shams et al. 2011; Boyd 1988), central nerve system (CNS) depressant (Drews 2000), antibacterial (Maren 1976; Supuran and Scozzafava 2000), anticancer (Simunek et al. 2007), anti-inflammatory, and antimicrobial (Abbate et al. 2004; Abdel-Mohsen and Hussein 2014). Moreover, Mannich bases are reported to show a variety of biological activities, such as antibacterial and antifungal activities (Singare and Ingle 1976; Huneck et al. 1993; Hussein et al. 2015a). Based on these prior observations, we postulated that a Schiff base containing both indoline and pyran pharmacophores could be very effective for antimicrobial and anticancer activity. In this paper and as a consequence of our previous work on the green synthesis of different spiroheterocyclic (Hussein 2013; Hussein and El-Khawaga 2012; Hussein 2012; El-Zohry et al. 2008b, c, 2009), and bioactive heterocyclic compounds (Hussein et al. 2015b; Hussein and Abdel-Monem 2011), we investigated a novel green and efficient protocol that was developed for the synthesis of some Schiff bases (**5a**–**l**) by the condensation of spiro[indoline-3,4′-pyran]-3′-carbonitrile derivatives (**3a**–**c**) with aromatic aldehydes (**4a**–**d**) using ammonium chloride (10 mol%) in refluxing ethanol as shown in Scheme 2 and Table 1. The antimicrobial and cytotoxic properties of the prepared compounds were screened.

**Table 1:** Synthesis of the Schiff bases **5a**–**l** using NH_4_Cl (10 mol%)

Entry	Product^a^	R_1_, R_2_	R_3_	Yield^b^ (%)
1	**5a**	(C_6_H_5_)_2_	2-OH	92
2	**5b**	(C_6_H_5_)_2_	4-OCH_3_	82
3	**5c**	(C_6_H_5_)_2_	4-Cl	78
4	**5d**	(C_6_H_5_)_2_	4-NO_2_	75
5	**5e**	(C_2_H_5_)_2_	2-OH	88
6	**5f**	(C_2_H_5_)_2_	4-OCH_3_	84
7	**5g**	(C_2_H_5_)_2_	4-Cl	80
8	**5h**	(C_2_H_5_)_2_	4-NO_2_	77
9	**5i**	1-Piperidinyl	2-OH	90
10	**5j**	1-Piperidinyl	4-OCH_3_	86
11	**5k**	1-Piperidinyl	4-Cl	84
12	**5l**	1-Piperidinyl	4-NO_2_	74

^a^Reaction conditions: spiro[indoline-3,4′-pyran]-3′-carbonitrile derivatives **3a**–**c** (10 mmol), aromatic aldehydes **4a**–**d** (10 mmol), and NH_4_Cl (10 mol%) in 10 mL ethanol/reflux, 2 h

^b^Isolated yields

## Results and discussion

### Chemistry

#### Synthesis of spiro[indoline-3,4′-pyran]-3′-carbonitrile derivatives (3a–c)

**Scheme 1:**
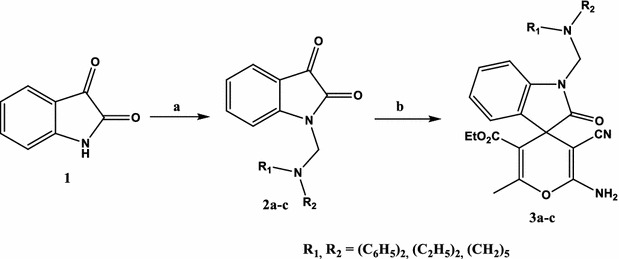
Synthesis of spiro[indoline-3,4′-pyran]-3′-carbonitrile derivatives **3a**–**c**. Reagents and conditions: *a* Mannich reaction (HCHO and secondary amine in ethanol, rt, 2–4 h, *b* malononitrile and ethyl acetoacetate in ethanol/piperidine (one drop), reflux, 5–6 h

The spiro[indoline-3,4′-pyran]-3′-carbonitrile derivatives **3a**–**c** described in this study were prepared as outlined in Scheme 1. The isatin Mannich bases **2a**–**c** were prepared by condensing the active hydrogen atom of istain with formaldehyde and secondary amine namely diphenylamine, diethylamine, and piperidine in ethanol at room temperature as previously reported procedure (Solomon et al. 2009). Compounds **3a**–**c** were obtained in good yield via three-component condensation of **2a**–**c**, malononitrile and ethyl acetoacetate in refluxed ethanol in presence of catalytic amount of piperidine.

#### Synthesis of target compounds

**Scheme 2:**
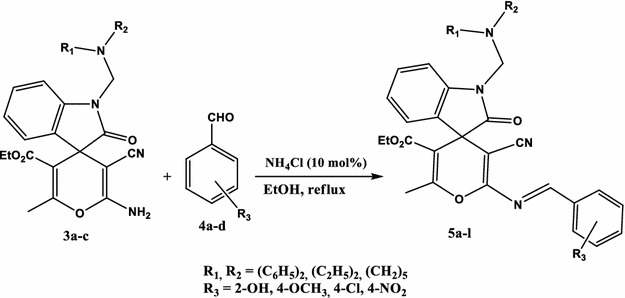
Synthesis of Schiff bases **5a**–**l**

The Schiff bases **5a**–**l** were obtained by the condensation of spiro[indoline-3,4′-pyran]-3′-carbonitrile derivatives **3a**–**c** with aromatic aldehydes **4a**–**d** using ammonium chloride (10 mol%) in refluxing ethanol (Scheme 2; Table 1).

**Scheme 3:**
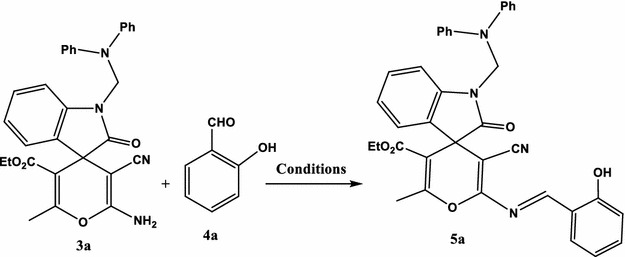
Model reaction

To find out the suitable conditions for the reaction, a series of experiments were performed with the standard reaction of ethyl 2′-amino-3′-cyano-1-((diphenylamino)methyl)-6′-methyl-2-oxospiro[indoline-3,4′-pyran]-5′-carboxylate (**3a**), salicylaldehyde (**4a**) as a model reaction (Table 2; Scheme 3).

**Table 2:** The effect of reaction condition on the synthesis of **5a**

Entry	Solvent^a^	Catalyst^b^	Yield^c^ (%)
1	AcOH	–	47
2	MeOH	–	40
3	EtOH	–	44
4	DMF	AcOH	52
5	EtOH	AcOH	61
6	EtOH	Et_3_N	71
7	EtOH	Piperidine	71
8	Dioxane	NH_4_Cl	73
9	DMF	NH_4_Cl	75
10	MeOH	NH_4_Cl	87
11	EtOH	NH_4_Cl	92

The reaction was carried out with ethyl 2′-amino-3′-cyano-1-((diphenylamino)methyl)-6′-methyl-2-oxospiro[indoline-3,4′-pyran]-5′-carboxylate (**3a**) (10 mmol) and 2-hydroxybenzaldehydes (**4a**) (10 mmol)

^a^10 mL solvent/reflux, 2 h

^b^10 mol%

^c^Isolated yields

#### Effect of the reaction conditions

In our initial study, we tried to optimize the model procedure mentioned above by detecting the efficiency of different reaction conditions in the absence and presence of catalysts, such as AcOH, MeOH, EtOH, DMF/AcOH, EtOH/AcOH, EtOH/Et_3_N, EtOH/piperidine, dioxane/NH_4_Cl, DMF/NH_4_Cl, MeOH/NH_4_Cl, and EtOH/NH_4_Cl (Scheme 3).

In each case, the reactants (10 mmol) were allowed together in 10 mL solvent at reflux temperature for 2 h. In the absence of catalyst, the reaction proceeded with comparatively lower reaction yield (Table 2, entries 1–3). DMF/AcOH, EtOH/AcOH, EtOH/Et_3_N and EtOH/piperidine can push the reaction towards the formation of product in yields of 52, 61, 71, and 71 %, respectively (Table 2, entries 4–7). In the presence of ammonium chloride (NH_4_Cl) the reaction was possible and the product (**5a**) was obtained in good yields. Ammonium chloride was used in different reaction media such as dioxane, DMF, methanol and ethanol (Table 2, entries 8–11). The best results were obtained when NH_4_Cl was used as catalyst in ethanol as reaction medium, which provided a yield of 92 %.

#### Evaluation of catalytic activity of ammonium chloride

To determine the appropriate concentration of the catalyst used, we investigated the model reaction at different concentrations of NH_4_Cl (5, 10, 15, 20, and 25 mol%). The product was formed in 80, 92, 92, 89, and 85 % yield, respectively (Table 3). This indicates that 10 mol% NH_4_Cl is sufficient to carry out the reaction smoothly.

**Table 3:** Evaluation of catalytic activity of NH_4_Cl in the synthesis of **5a**

Entry	Amount of NH_4_Cl (mol%)	Yield^a^ (%)
1	5	80
2	10	92
3	15	92
4	20	89
5	25	85

The reaction was carried out with ethyl 2′-amino-3′-cyano-1-((diphenylamino)methyl)-6′-methyl-2-oxospiro[indoline-3,4′-pyran]-5′-carboxylate (**3a**) (10 mmol) and 2-hydroxybenzaldehydes (**4a**) (10 mmol), and NH_4_Cl in 10 mL ethanol at refluxing temperature/2 h

^a^Isolated yield of (**5a**)

The structures of the isolated new products **5a–l** were deduced by analyzing their physical and spectroscopic data, such as the data obtained using IR, ^1^H NMR, and ^13^C NMR spectroscopy. Taking **5a** as an example, broad absorption band at 3356 cm^−1^ for OH group, sharp absorption band at 2210 cm^−1^ for CN group, and two absorption band at 1735, 1620 cm^−1^ for two C=O groups were observed in the IR spectrum with absence of absorption bands at 3350, 3260 cm^−1^ which corresponding to NH_2_ group. The ^1^H NMR spectrum showed the presence of triplet and quartet signals at 1.28, and 3.85 for ethyl protons, as well as, four singlet signals at δ = 2.28, 5.30, 8.15, and 10.38 ppm for the methyl, methylene, methane, and OH protons, respectively. In the ^13^C NMR spectrum, the quaternary spiro carbon typically appeared at δ = 48.9 ppm. The nitrile and two carbonyl carbons resonated at 117.4, 164.4, and 178.5 ppm, respectively.

### Biological activity

#### Antimicrobial activity

In view of biological significance, it was studied the synthesized some spiro-indoline derivatives as previous, to get the activities of the potent compounds and evaluated their potential in vitro as antibacterial, antifungal and antitumor activities.

Antimicrobial activities of all the synthesized Schiff bases **5a**–**l** were done by cup-plate agar diffusion method. The compounds were prepared in DMSO and evaluated them for their in vitro antibacterial and antifungal activities against *Bacillus subtilis* and *Fusarium moniliforme* respectively. The bacterial isolate was grown on nutrient agar (37 °C, 24 h) the fungus was grown on potato dextrose agar plates (26 °C, 48–72 h). The results were noted by the presence of clear zone of inhibition around the active compounds (Table 4).

**Table 4:** Biological activities of the synthesized spiro-indoline derivatives **3a**–**c** and **5a**–**l**

Compounds	R_1_, R_2_	R_3_	IC_50_	Inhibition zone (mm) *B. subtilis*	Inhibition zone (mm) *F. moniliforme*
**3a**	(C_6_H_5_)_2_	–	>50	12 ± 2	Nill
**3b**	(C_2_H_5_)_2_	–	>50	24 ± 2	15 ± 2
**3c**	Piperidinyl	–	18.8	19 ± 4	2 ± 1
**5a**	(C_6_H_5_)_2_	2-OH	44.5	20 ± 2	12 ± 1
**5b**	(C_6_H_5_)_2_	4-OCH_3_	23.9	Nill	Nill
**5c**	(C_6_H_5_)_2_	4-Cl	>50	24 ± 2	10 ± 2
**5d**	(C_6_H_5_)_2_	4-NO_2_	25.0	18 ± 3	6 ± 1
**5e**	(C_2_H_5_)_2_	2-OH	25.0	18 ± 1	4 ± 1
**5f**	(C_2_H_5_)_2_	4-OCH_3_	>50	Nill	Nill
**5g**	(C_2_H_5_)_2_	4-Cl	11.9	Nill	Nill
**5h**	(C_2_H_5_)_2_	4-NO_2_	20.9	25 ± 3	10 ± 1
**5i**	Piperidinyl	2-OH	>50	19 ± 3	Nill
**5j**	Piperidinyl	4-OCH_3_	18.8	16 ± 2	4 ± 2
**5k**	Piperidinyl	4-Cl	>50	12 ± 1	Nill
**5l**	Piperidinyl	4-NO_2_	38.3	4 ± 1	Nill

All the synthesized compounds **3a**–**c** and **5a**–**l** were tested for in vitro antibacterial activity by inhibition zone method against the reference compound amoxicillin (20 mm). It has been observed that all the compounds tested showed mild to moderate activity against tested bacterium but **5f**, **5g** and **3a**. The antifungal activity of the compounds was studied with *F. Moniliforme*. The results are summarized in Table 4. Fluconazole has been used as reference for inhibitory activity (18 mm) against fungi and some tested compounds showed lesser activity to standard against the tested fungi. While the others showed no antifungal activities against the fungus.

#### In vitro anticancer activity

Antitumor activities were found moderate effective as screened for in vitro cytotoxicity activity against human cancer cells line MCF7 breast cancer (Table 5). Although the positive impact of each of the synthesized compound conducted toxicity in cells, some lost IC_50_ in the concentrations used. Viewing of the results, the IC_50_ required was higher than that of the reference compound (3.8 µg/mL) used in the analysis. There are no significant differences between the results of the synthetic chemical compounds compared to the reference compound, where statistically significant differences is the numerical value, and therefore all synthesized compounds located with reference drug in one hand.

**Table 5:** Cytotoxicity activity of spiro-indoline derivatives **3a**–**c** and **5a**–**l** at different concentrations (0.0, 5.0, 12.5, 25 and 50 µg/mL) of some synthesized compounds and reference drug against human cancer cells line MCF7 breast cancer

CONC	DOX	**3a**	**5a**	**5b**	**5c**	**5d**	**3b**	**5e**	**5f**	**5g**	**5h**	**3c**	**5i**	**5j**	**5k**	**5l**
0.0	1.0	1.0	1.0	1.0	1.0	1.0	1.0	1.0	1.0	1.0	1.0	1.0	1.0	1.0	1.0	1.0
5.0	0.361	0.836	0.758	0.867	0.930	0.977	0.786	0.911	0.764	0.853	0.978	0.799	0.974	0.688	0.824	0.906
12.5	0.385	0.738	0.479	0.633	0.837	0.740	0.542	0.876	0.643	0.728	0.669	0.642	0.752	0.637	0.588	0.803
25.0	0.332	0.653	0.340	0.498	0.698	0.619	0.462	0.467	0.547	0.560	0.628	0.437	0.829	0.498	0.414	0.725
50.0	0.299	0.631	0.353	0.502	0.442	0.619	0.444	0.416	0.608	0.453	0.564	0.526	0.772	0.479	0.368	0.829
SD	0.024	0.009	0.013	0.025	0.020	0.016	0.034	0.039	0.120	0.022	0.020	0.025	0.010	0.013	0.058	0.009

It is worth mentioning, that the curve of the compound **5c** only showed clear a straight line. A high concentration compared to the control has shown. Theoretically an expectation of the IC_50_ may be located on the curve. Statistically the range of lethal concentrations IC_50_ may be at about 70 µg/mL, concentration that’s when kills ninety percent of the living cells.

**Fig. 1:**
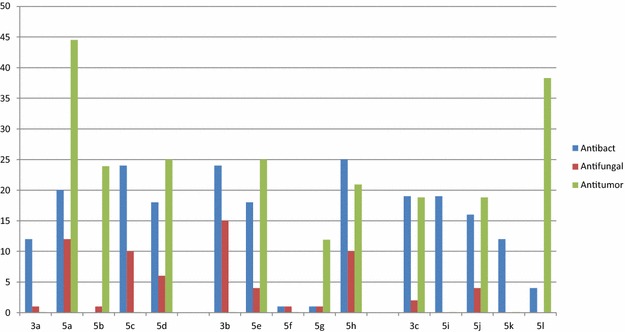
Biological activities of spiro-indoline derivatives of **3a**–**c** and **5a**–**l**

The biological activities of **3a**–**c** and its derivatives **5a**–**l** were summarized in (Fig. 1). Only cell toxicity of **3a** did not record (>50 µg/mL) and antimicrobial activities of **5b** and antifungal activity of **5c** were not detected. The compounds **3b** and **5f** just showed IC_50_ exceed 50 µg/mL but antimicrobial activities did not detect by **5g** and **5f** only. IC_50_ did not show in the tested concentrations of **5i** and **5k**. Also antifungal activities did not record in **5i**, **5k** and **5l** as shown in (Fig. 1).

### Conclusion

The authors have developed a green, rapid and efficient protocol for the synthesis of a new series of Schiff bases from spiro[indoline-3,4′-pyran]-3′-carbonitrile derivatives using ammonium chloride as a very inexpensive and readily available reagent. The prepared compounds were assessed in vitro for their antibacterial activity against *B. subtilis* as well as antifungal activity against *F. moniliforme.* Also, the cytotoxic activity of the prepared compounds was assessed in vitro against human cells line MCF7 breast cancer.

## Experimental

### Chemistry

#### General methods

The IR spectra of the synthesized compounds were taken on a Shimazu FT spectrometer with a device of singly perturbed internal reflection. ^1^HNMR spectra (in DMSO-d_6_) were recorded on Bruker Ac-400 ultra-shield NMR spectrometer at 400 MHz, using TMS as internal standard. The ^13^C NMR (100 MHz) spectra were run in dimethylsulfoxide (DMSO-d_6_). Chemical shifts were related to that of the solvent. Mass spectra were obtained on a Joel JMSD-300 spectrometer operating at 70 eV. The elemental analysis was carried out on a perkin-Elmer C, H, N analyzer. Melting points were determined in open capillaries on a Gallenkemp melting point apparatus and are uncorrected.

- Synthesis of spiro[indoline-3,4′-pyran]-3′-carbonitrile derivatives **3a**–**c**

##### General procedure

A mixture of 1-((diphenylamino)methyl)indoline-2,3-dione (**2a**) (3.28 g, 10 mmol) and malononitrile (0.66 g, 10 mmol) was dissolved in 20 mL absolute ethanol and stirred for 30 min. Then ethyl acetoacetate (1.30 g, 10 mmol) was added in the presence of piperidine (one drop) and the reaction mixture was heated under reflux with stirring for 6 h. Then cooled and the formed crystals was collected by filtration. Dried and recrystallized for a proper solvent.

##### Ethyl-6-amino-5-cyano-1′-((diphenylamino)methyl)-2-methyl-2′-oxo-4*H*-spiro[pyran-4,3′-indoline]-3-carboxylate (**3a**)

White crystals (ethanol), yield 75 %, mp 225–227 °C. IR (KBr): 3260, 3150 (NH_2_), 2185 (CN), 1724 (C=O), 1670 (C=O). ^1^H NMR: δ = 1.25 (t, 3H, CH_3_), 2.29 (s, 3H, CH_3_), 3.98–4.00 (q, 2H, CH_2_), 5.41 (s, 2H, CH_2_), 6.80 (s, 2H, NH_2_, D_2_O-exchangeable), 6.78–7.71 (m, 14H, Ar–H) ppm. ^13^C NMR: δ = 13.6 (CH_3_), 18.6 (CH_3_), 49.0 (C-spiro), 56.57, 60.3 (CH_2_), 76.7 (CH_2_), 100.4, 118.5 (CN), 121.5, 121.9, 123.0, 123.4, 125.1, 128.6, 128.7, 142.1, 151.0, 156.5, 159.2, 164.6 (C=O), 166.7 (C=O) ppm. MS: *m*/*z* (%) = 506.05 (M^+^, 45), 169.11 (100). Anal. Calcd. For C_30_H_26_N_4_O_4_ (506.55): C, 71.13; H, 5.17; N, 11.06. Found: C, 71.17; H, 5.08; N, 10.89.

##### Ethyl-6-amino-5-cyano-1′-((diethylamino)methyl)-2-methyl-2′-oxo-4*H*-spiro[pyran-4,3′-indoline]-3-carboxylate (**3b**)

As pale yellow crystals (dioxane), yield 90 %, mp 140–142 °C. IR (KBr): 3270, 3190 (NH_2_), 2190 (CN), 1722 (C=O), 1660, (C=O). ^1^H NMR: δ = 1.10 (t, 6H, CH_3_), 1.22 (t, 3H, CH_3_), 1.72 (s, 3H, CH_3_), 2.48 (q, 4H, 2CH_2_), 4.18–4.20 (q, 2H, OCH_2_), 4.39 (s, 2H, CH_2_), 6.79 (s, 2H, NH_2_, D_2_O-exchangeable), 6.92–7.76 (m, 4H, Ar–H) ppm. MS: *m*/*z* (%) = 410.19 (M^+^, 23), 133 (100). Anal. Calcd. For C_22_H_26_N_4_O_4_ (410.47): C, 64.37; H, 6.38; N, 13.65. Found: C, 64.42; H, 6.37; N, 13.59.

##### Ethyl-6-amino-5-cyano-1′-(piperidin-1-ylmethyl)-2-methyl-2′-oxo-4*H*-spiro[pyran-4,3′-indoline]-3-carboxylate (**3c**)

As pale yellow crystals (ethanol), yield 87 %, mp 189–190 °C. IR (KBr): 3240, 3100 (NH_2_), 2170 (CN), 1715 (C=O), 1665 (C=O). ^1^H NMR: δ = 1.30 (t, 3H, CH_3_), 1.57–1.59 (m, 6H, 3CH_2_), 1.74 (s, 3H, CH_3_), 2.60 (t, 4H, 2CH_2_), 4.19–4.21 (q, 2H, CH_2_), 4.31 (s, 2H, CH_2_), 6.85 (s, 2H, NH_2_, D_2_O-exchangeable), 6.87–7.26 (m, 4H, Ar–H) ppm. ^13^C NMR: δ = 13.9 (CH_3_), 14.1 (CH_3_), 26.0, 26.3, 48.1 (C-spiro), 52.8 (C-pipredine), 53.9, 61.6 (CH_2_), 76.8 (CH_2_), 106.1, 120.0 (CN), 122.9, 123.8, 126.5, 127.5, 131.2, 138.0, 151.5, 156.2, 165.44 (C=O), 167.3 (C=O). MS: *m*/*z* (%) = 422.15 (M^+^, 23), 142 (100). Anal. Calcd. For C_23_H_26_N_4_O_4_ (422.48): C, 65.39; H, 6.20; N, 13.26. Found: C, 65.36; H, 6.18; N, 13.19.

- General procedure for the synthesis of the Schiff bases **5a–l**

##### General procedure

To a solution of spiro[indoline-3,4′-pyran]-3′-carbonitrile derivative 3a (0.51 g, 1 mmol) in absolute ethanol (10 mL), corresponding aromatic aldehyde (1 mmol) was added. Then NH_4_Cl (5.35 mg, 10 mol %) was added and the reaction mixture was refluxed for 2 h (monitored by TLC). After completion of the reaction, cold water (15–25 mL) was added to the reaction mixture. The solid product was filtered, washed with cold water, dried, and recrystallized from proper solvents.

##### Ethyl-6-(2-hydroxybenzylidenamino)-5-cyano-1′-((diphenylamino)methyl)-2methyl-2′-oxo-4*H*-spiro[pyran-4,3′-indoline]-3-carboxylate (**5a**)

As yellow crystals (ethanol), mp 215–217 °C. IR (KBr): 3356 (br. OH), 2210 (CN), 1735 (C=O), 1620 (C=O). ^1^H NMR (DMSO-d_6_): δ = 0.85 (t, 3H, CH_3_), 2.53 (s, 3H, CH_3_), 3.80 (s, 2H, CH_2_), 3.82–3.85 (q, 2H, CH_2_), 6.80–7.21 (m, 18H, Ar–H), 10.27 (s, 1H, OH), 10.38 (s, 1H, N=CH) ppm. ^13^C NMR (DMSO-d_6_): δ = 12.9 (CH_3_), 18.5 (CH_3_), 48.9 (C-spiro), 60.2 (CH_2_), 74.9 (CH_2_), 104.6, 111.0, 117.4 (CN), 123.3, 125.7, 127.6, 127.9, 128.4, 129.0, 131.1, 131.6, 134.5, 142.1, 144.2, 158.4, 158.9, 163.7 (N=CH), 164.4 (C=O), 178.5 (C=O). MS: *m*/*z* (%) = 610.08 (M^+^, 10), 262.10 (100). Anal. Calcd. For C_37_H_30_N_4_O_5_ (610.66): C, 72.77; H, 4.95; N, 9.17. Found: C, 72.82; H, 4.76; N, 9.14.

##### Ethyl-6-(4-methoxybenzylidenamino)-5-cyano-1′-((diphenylamino)methyl)-2-methyl-2′-oxo-4*H*-spiro[pyran-4,3′-indoline]-3-carboxylate (**5b**)

As pale yellow crystals (ethanol), mp 212–214 °C. IR (KBr): 2180 (CN), 1740 (C=O), 1625 (C=O). ^1^H NMR (DMSO-d_6_): δ = 0.80 (t, 3H, CH_3_), 2.33 (s, 3H, CH_3_), 3.32 (s, 3H, OCH_3_), 3.81–3.83 (q, 2H, CH_2_), 3.89 (s, 2H, CH_2_), 6.80–7.90 (m, 18H, Ar–H), 10.38 (s, 1H, N=CH) ppm. ^13^C NMR (DMSO-d_6_): δ = 12.9 (CH_3_), 18.5 (CH_3_), 48.9 (C-spiro), 55.6 (CH_3_), 56.6, 60.2 (CH_2_), 75.4 (CH_2_), 104.6, 117.4 (CN), 119.3, 121.5, 122.9, 123.8, 125.1, 127.9, 128.4, 129.1, 130.3, 131.7, 134.5, 142.1 (C-aromatic), 158.3 (C-pyrane), 164.6 (N=CH), 169.4 (C=O), 178.5(C=O). MS: *m*/*z* (%) = 624.20 (M^+^, 13), 252.51 (100). Anal. Calcd. For C_38_H_32_N_4_O_5_ (624.68): C, 73.06; H, 5.16; N, 8.97. Found: C, 72.91; H, 4.90; N, 9.02.

##### Ethyl-6-(4-chlorobenzylidenamino)-5-cyano-1′-((diphenylamino)methyl)-2-methyl-2′-oxo-4*H*-spiro[pyran-4,3′-indoline]-3-carboxylate (**5c**)

As pale brown crystals (ethanol), mp 230–232 °C. IR (KBr): 2180 (CN), 1742 (C=O), 1635 (C=O). ^1^H NMR (DMSO-d_6_): δ = 0.80 (t, 3H, CH_3_), 2.33 (s, 3H, CH_3_), 3.80 (s, 2H, CH_2_), 3.82–3.85 (q, 2H, CH_2_), 6.80–7.71 (m, 18H, Ar–H), 10.38 (s, 1H, N=CH) ppm. MS: *m*/*z* (%) = 628.61 (M^+^, 16), 262.11 (100). Anal. Calcd. For C_37_H_29_ClN_4_O_4_ (629.10): C, 70.64; H, 4.65; Cl, 5.64; N, 8.91. Found: C, 70.75; H, 4.48; Cl, 5.50; N, 8.93.

##### Ethyl-6-(4-nitrobenzylidenamino)-5-cyano-1′-((diphenylamino)methyl)-2-methyl-2′-oxo-4*H*-spiro[pyran-4,3′-indoline]-3-carboxylate (**5d**)

As pale yellow crystals (ethanol), mp 235–237 °C. IR (KBr): 2200 (CN), 1740 (C=O), 1630 (C=O). ^1^H NMR (DMSO-d_6_): δ = 0.80 (t, 3H, CH_3_), 2.30 (s, 3H, CH_3_), 3.32 (s, 2H, CH_2_), 3.81–3.83 (q, 2H, CH_2_), 6.80–8.24 (m, 18H, Ar–H), 10.37 (s, 1H, N=CH) ppm. ^13^C NMR (DMSO-d_6_): δ = 12.90 (CH_3_), 18.46 (CH_3_), 48.91 (C-spiro), 61.12 (CH_2_), 75.48 (CH_2_), 104.64, 117.35 (CN), 118.45, 121.74, 123.35, 125.75, 127.95, 128.52, 129.21, 131.77, 134.47, 142.00, 158.87 (C-pyrane), 163.65 (N=CH), 166.41 (C=O), 178.45 (C=O). MS: *m*/*z* (%) = 639.20 (M^+^, 13), 169.69 (100). Anal. Calcd. For C_37_H_29_N_5_O_6_ (639.66): C, 69.47; H, 4.57; N, 10.95. Found: C, 69.44; H, 4.48; N, 10.81.

##### Ethyl-6-(2-hydroxybenzylidenamino)-5-cyano-1′-((diethylamino)methyl)-2-methyl-2′-oxo-4*H*-spiro[pyran-4,3′-indoline]-3-carboxylate (**5e**)

As pale yellow crystals (ethanol), mp 125–127 °C. IR (KBr): 3414 (br. OH), 2191 (CN), 1724 (C=O), 1620 (C=O). ^1^H NMR (DMSO-d_6_): δ = 1.15 (t, 6H, 2CH_3_), 1.24 (t, 3H, CH_3_), 2.27 (s, 3H, CH_3_), 2.39 (q, 4H, 2CH_2_), 3.86 (s, 2H, CH_2_), 4.32–4.39 (m, 4H, 2CH_2_), 6.84–7.80 (m, 8H, Ar–H), 9.85 (s, 1H, N=CH), 12.50 (s, 1H, OH, exchangeable with D_2_O) ppm. ^13^C NMR (DMSO-d_6_): δ = 13.8 (2CH_3_), 14.1 (CH_3_), 14.2 (CH_3_), 49.0 (C spiro), 61.4 (2CH_2_), 61.6 (CH_2_), 67.7 (CH_2_), 100.2, 110.9 (CN), 123.1, 123.4, 123.7, 124.3, 125.7, 127.7, 129.2, 130.4, 131.2, 134.6, 140.1, 147.8, 162.2, 166.3 (N=CH), 167.3 (C=O), 170.3 (C=O). MS: *m*/*z* (%) = 514.08 (M^+^, 41), 262.14 (100). Anal. Calcd. For C_29_H_30_N_4_O_5_ (514.57): C, 67.69; H, 5.88; N, 10.89. Found: C, 67.42; H, 5.62; N, 11.01.

##### Ethyl-6-(4-methoxybenzylidenamino)-5-cyano-1′-((diethylamino)methyl)-2-methyl-2′-oxo-4*H*-spiro[pyran-4,3′-indoline]-3-carboxylate (**5f**)

As pale yellow crystals (petroleum ether 60–80), mp 70–72 °C. IR (KBr): 2160 (CN), 1720 (C=O), 1650 (C=O). ^1^H NMR (DMSO-d_6_): δ = 0.84–1.24 (m, 6H, 2CH_3_), 1.41 (t, 3H, CH_3_), 2.83 (s, 3H, CH_3_), 3.86 (s, 3H, OCH_3_), 4.19–4.39 (m, 8H, 3CH_2_), 6.85–7.83 (m, 8H, Ar–H), 9.87 (s, 1H, N=CH) ppm. MS: *m*/*z* (%) = 528.21 (M^+^, 20), 234.05 (100). Anal. Calcd. For C_30_H_32_N_4_O_5_ (528.60): C, 68.17; H, 6.10; N, 10.60. Found: C, 68.21; H, 6.01; N, 10.56.

##### Ethyl-6-(4-chlorobenzylidenamino)-5-cyano-1′-((diethylamino)methyl)-2-methyl-2′-one-4*H*-spiro[pyran-4,3′-indoline]-3-carboxylate (**5g**)

As pale yellow crystals (diethyl ether), mp 90–92 °C. IR (KBr): 2190 (CN), 1730 (C=O), 1655 (C=O). ^1^H NMR (DMSO-d_6_): δ = 0.91–1.32 (m, 6H, 2CH_3_), 1.39 (t, 3H, CH_3_), 2.83 (s, 3H, CH_3_), 4.19–4.39 (m, 6H, 3CH_2_), 5.51 (s, 2H, CH_2_), 7.03–7.65 (m, 8H, Ar–H), 9.88 (s, 1H, N=CH) ppm. MS: *m*/*z* (%) = 532.09 (M^+^, 31), 146.00 (100). Anal. Calcd. For C_29_H_29_ClN_4_O_4_ (533.02): C, 65.35; H, 5.48; Cl, 6.65; N, 10.51. Found: C, 65.45; H, 5.73; Cl, 6.61; N, 10.47.

##### Ethyl-6-(4-nitrobezylidenamino)-5-cyano-1′-((diethylamino)methyl)-2-methyl-2′-oxo-4*H*-spiro[pyran-4,3′-indoline]-3-carboxylate (**5h**)

As pale yellow crystals (diethyl ether), m.p 85–87 °C. IR (KBr): 2170 (CN), 1724 (C=O), 1650 (C=O). ^1^H NMR (DMSO-d_6_): δ = 0.88–1.31 (m, 6H, 2CH_3_), 1.41 (t, 3H, CH_3_), 2.83 (s, 3H, CH_3_), 4.21–4.36 (m, 6H, 3CH_2_), 4.38 (s, 2H, CH_2_), 7.12–7.85 (m, 8H, Ar–H), 9.89 (s, 1H, N=CH) ppm. ^13^C NMR (DMSO-d_6_): δ = 13.9 (2CH_3_), 14.2 (CH_3_), 15.1 (CH_3_), 49.1 (C spiro), 61.3 (2CH_2_), 61.6 (CH_2_), 76.7 (CH_2_), 100.1, 117.3 (CN), 123.1, 123.4, 123.7, 124.3, 125.7, 127.7, 129.2, 130.4, 131.2, 134.6, 147.9, 162.2, 166.0 (N=CH), 167.3 (C=O), 170.0 (C=O). MS: *m*/*z* (%) = 543.01 (M^+^, 15), 234 (100). Anal. Calcd. For C_29_H_29_N_5_O_6_ (543.57): C, 64.08; H, 5.38; N, 12.88. Found: C, 64.04; H, 5.64; N, 12.84.

##### Ethyl-6-(2-hydroxybenzylidenamino)-5-cyano-1′-(piperidin-1-ylmethyl)-2methyl-2′-oxo-4*H*-spiro[pyran-4,3′-indoline]-3-carboxylate (**5i**)

As pale yellow crystals (diethyl ether), mp 110–112 °C. IR (KBr): 3455 (br. OH), 2210 (CN), 1753 (C=O), 1634 (C=O). ^1^H NMR (DMSO-d_6_): δ = 090–1.13 (m, 6H, 3CH_2_), 1.16 (t, 3H, CH_3_), 2.67 (t, 4H, 2CH_2_), 3.46 (s, 3H, CH_3_), 3.64 (s, 2H, CH_2_), 4.15–4.31 (q, 2H, CH_2_), 6.81–7.87 (m, 8H, Ar–H), 9.96 (s, 1H, N=CH), 10.51 (s, 1H, OH) ppm. ^13^C NMR (DMSO-d_6_): δ = 13.9 (CH_3_), 14.1 (CH_3_), 26.2, 26.6, 52.7 (CH_2_-piperidine), 49.0 (C-spiro), 53.9, 61.1, 61.6 (CH_2_), 76.7 (CH_2_), 109.2, 117.6 (CN), 119.8, 120.1, 121.5, 123.0, 123.8, 124.6, 126.5, 131.1, 133.7, 137.0, 140.6 (C-aromatic), 156.9, 163.5 (N=CH), 165.4 (C=O), 167.2 (C=O). MS: *m*/*z* (%) = 526.09 (M^+^, 5), 234.22 (100). Anal. Calcd. For C_30_H_30_N_4_O_5_ (526.58): C, 68.43; H, 5.74; N, 10.64. Found: C, 68.40; H, 5.71; N, 10.36.

##### Ethyl-6-(4-methoxybenzylidenamino)-5-cyano-1′-(piperidin-1-ylmethyl)-2-methyl-2′-oxo-4*H*-spiro[pyran-4,3′-indoline]-3-carboxylate (**5j**)

As brown crystals (diethyl ether), mp 114–117 °C. IR (KBr): 2210 (CN), 1742 (C=O), 1631 (C=O). ^1^H NMR (DMSO-d_6_): δ = 0.90–1.13 (m, 6H, 3CH_2_), 1.16 (t, 3H, CH_3_), 2.67 (t, 4H, 2CH_2_), 3.46 (s, 3H, CH_3_), 3.64 (s, 2H, CH_2_), 3.75 (s, 3H, OCH_3_), 4.15–4.30 (q, 2H, CH_2_), 6.86–7.89 (m, 8H, Ar–H), 9.95 (s, 1H, N=CH) ppm. ^13^C NMR (DMSO-d_6_): δ = 14.0 (CH_3_), 14.1 (CH_3_), 25.9, 26.1 (CH_2_), 48.4 (C-spiro), 52.1 (CH_2_), 53.4 (CH_3_), 55.8, 61.6 (CH_2_), 76.8 (CH_2_), 106.0, 120.0 (CN), 121.1, 122.6, 123.3, 124.8, 125.9, 126.3, 128.3, 129.7, 131.9, 156.2, 164.3, 164.7 (N=CH), 167.5 (C=O), 169.9 (C=O). MS: *m*/*z* (%) = 540.20 (M^+^, 31), 299 (100). Anal. Calcd. For C_31_H_32_N_4_O_5_ (540.61): C, 68.87; H, 5.97; N, 10.36. Found: C, 68.63; H, 5.69; N, 10.32.

##### Ethyl-6-(4-chlorobenzylidenamino)-5-cyano-1′-(piperidin-1-ylmethyl)-2methyl-2′-oxo-4*H*-spiro[pyran-4,3′-indoline]-3-carboxylate (**5k**)

As pale yellow crystals (diethyl ether), mp 140–142 °C. IR (KBr): 2219 (CN), 1725 (C=O), 1622 (C=O). ^1^H NMR (DMSO-d_6_): δ = 1.13–1.17 (m, 6H, 3CH_2_), 1.25 (t, 3H, CH_3_), 2.65 (t, 4H, 2CH_2_), 3.46 (s, 3H, CH_3_), 3.65 (s, 2H, CH_2_), 4.15–4.31 (q, 2H, CH_2_), 6.81–7.85 (m, 8H, Ar–H), 9.96 (s, 1H, N=CH) ppm. ^13^C NMR (DMSO-d_6_): δ = 14.1 (CH_3_), 14.2 (CH_3_), 26.3, 26.6 (CH_2_), 49.1 (C-spiro), 53.9 (CH_2_), 61.3, 61.6 (CH_2_), 76.7 (CH_2_), 106.2, 120.0 (CN), 121.5, 123.0, 124.5, 125.7, 126.5, 128.3, 128.7, 130.9, 131.1, 134.7 (C-aromatic), 156.2, 163.7, 165.7 (N=CH), 167.4 (C=O), 174.1 (C=O). MS: *m*/*z* (%) = 544.10 (M^+^, 11), 261.15 (100). Anal. Calcd. For C_30_H_29_ClN_4_O_4_ (544.03): C, 66.11; H, 5.36; Cl, 6.50; N, 10.28. Found: C, 66.13; H, 5.30; Cl, 6.47; N, 10.32.

##### Ethyl-6-(4-nitrobenzylidenamino)-5-cyano-1′-(piperidin-1-ylmethyl)-2methyl-2′-oxo-4*H*-spiro[pyran-4,3′-indoline]-3-carboxylate (**5l**)

As pale brown crystals (ethanol), mp 160–162 °C. IR (KBr): 2210 (CN), 1732 (C=O), 1630 (C=O). ^1^H NMR (DMSO-d_6_): δ = 1.15–1.17 (m, 6H, 3CH_2_), 1.28 (t, 3H, CH_3_), 2.15 (t, 4H, 2CH_2_), 3.58 (s, 3H, CH_3_), 4.15–4.35 (q, 2H, CH_2_), 5.50 (s, 2H, CH_2_), 6.98–7.37 (m, 8H, Ar–H), 8.39 (s, 1H, N=CH) ppm. MS: *m*/*z* (%) = 555.12 (M^+^, 25), 205 (100). Anal. Calcd. For C_30_H_29_N_5_O_6_ (555.58): C, 64.85; H, 5.26; N, 12.61. Found: C, 64.82; H, 5.28; N, 12.59.

### Biological screening

#### Antibacterial activity

The newly synthesized spiro-indoline derivatives **3a**–**c** and **5a**–**l** were screened for their antibacterial activity against bacterial isolate namely *B. subtilis* by inhibition zone method against the reference compound amoxicillin (20 mm). The bacterial subcultures (18–24 h grown) were added to sterilize nutrient agar medium and shaken thoroughly to ensure uniform distribution of organism throughout the medium. In sterilized Petri dishes containing about 20 mL of the medium, wells were made with a sterile cork borer and were filled with 0.1 mL of respective solution. Then, the Petri dishes were kept for incubation in an inverted position for 24–48 h at 37 °C in an incubator. When growth inhibition zones were developed, diameter (in mm) was measured and compared with that of amoxicillin.

#### Antifungal activity

The newly synthesized spiro-indoline derivatives **3a**–**c** and **5a**–**l** were screened for their antifungal activity against fungus *F. moniliforme* at the concentration levels of 50 µg/mL by inhibition zone method. Fluconazole has been used as reference for inhibitory activity (18 mm) against fungi. To the sterilized potato dextrose agar medium, subculture of fungus were added and shaken thoroughly to ensure uniform distribution and incubated for 72 h. Then, this was poured into sterilized and labeled Petri dishes and allowed to solidify. Wells were made in each plate by a cork borer. Each well was filled with 0.1 mL of test solution and the other with respective concentrations of standard dilutions. The plates were left 2–3 h for diffusion and incubated at 37 °C for 24 h. The diameter of the zones of growth inhibition was measured and compared with that of standard. The solutions of required concentration (50 μg/mL) of test compounds were prepared by dissolving the compounds in DMSO.

#### Anticancer activity

Breast cancer cell line (MCF7) as human tumor was used in this study. The cytotoxicity was measured in vitro for the newly synthesized compounds assay using the method of Philip et al. (1990). The in vitro anticancer.

Screening was done by the pharmacology unit at Pharmacology unit, Cancer biology department, the National Cancer Institute, Cairo University. Cells were plated in 96-multiwell plate (104 cells/well) for 24 h before treatment with the compound(s) to allow attachment of cell to the wall of the plate. Tested compounds were dissolved in dimethyl sulfoxide (DMSO). Different concentrations of the compound under test (0.0, 5.0, 12.5, 25.0 and 50.0 µg/mL) were added to the cell monolayer. Triplicate wells were prepared for each individual concentration. Monolayer cells were incubated with the compound(s) for 48 h at 37 °C and in atmosphere of 5 % CO_2_. After 48 h. cells were fixed, washed and stained for 30 min with 0.4 % (W/V) SRB dissolved in 1 % acetic acid. Excess unbound dye was removed by four washes with 1 % acetic acid and attached stain was recovered with Tris–EDTA buffer. Color intensity was measured in an ELISA reader. The relation between surviving fraction and drug concentration is plotted to get the survival curve for breast tumor cell line after the specified time. The molar concentration required for 50 % inhibition of cell viability (IC_50_) was calculated and compared to the reference drug Doxorubicin (CAS, 25316-40-9). The surviving fractions were expressed as means and the results are given in Table 5.
